# Fibroblast Growth Factor-10 Promotes Cardiomyocyte Differentiation from Embryonic and Induced Pluripotent Stem Cells

**DOI:** 10.1371/journal.pone.0014414

**Published:** 2010-12-28

**Authors:** Sunny Sun-Kin Chan, Hui-Jing Li, Ying-Chang Hsueh, Desy S. Lee, Jyh-Hong Chen, Shiaw-Min Hwang, Chen-Yun Chen, Emily Shih, Patrick C. H. Hsieh

**Affiliations:** 1 Institute of Clinical Medicine and Research Center for Clinical Medicine, National Cheng Kung University and Hospital, Tainan, Taiwan; 2 Institute of Basic Medicine, National Cheng Kung University and Hospital, Tainan, Taiwan; 3 Department of Medicine, National Cheng Kung University and Hospital, Tainan, Taiwan; 4 Bioresource Collection and Research Center, Food Industry Research and Development Institute, Hsinchu, Taiwan; 5 Molecular Medicine Program, National Yang-Ming University, Taipei, Taiwan; 6 Institute of Biomedical Sciences, Academia Sinica, Taipei, Taiwan; 7 Department of Biochemistry, University of Washington, Seattle, Washington, United States of America; Cincinnati Children's Hospital Medical Center, United States of America

## Abstract

**Background:**

The fibroblast growth factor (FGF) family is essential to normal heart development. Yet, its contribution to cardiomyocyte differentiation from stem cells has not been systemically studied. In this study, we examined the mechanisms and characters of cardiomyocyte differentiation from FGF family protein treated embryonic stem (ES) cells and induced pluripotent stem (iPS) cells.

**Methodology/Principal Findings:**

We used mouse ES cells stably transfected with a cardiac-specific α-myosin heavy chain (αMHC) promoter-driven enhanced green fluorescent protein (EGFP) and mouse iPS cells to investigate cardiomyocyte differentiation. During cardiomyocyte differentiation from mouse ES cells, FGF-3, -8, -10, -11, -13 and -15 showed an expression pattern similar to the mesodermal marker Brachyury and the cardiovascular progenitor marker Flk-1. Among them, FGF-10 induced cardiomyocyte differentiation in a time- and concentration-dependent manner. FGF-10 neutralizing antibody, small molecule FGF receptor antagonist PD173074 and FGF-10 and FGF receptor-2 short hairpin RNAs inhibited cardiomyocyte differentiation. FGF-10 also increased mouse iPS cell differentiation into cardiomyocyte lineage, and this effect was abolished by FGF-10 neutralizing antibody or PD173074. Following Gene Ontology analysis, microarray data indicated that genes involved in cardiac development were upregulated after FGF-10 treatment. In vivo, intramyocardial co-administration of FGF-10 and ES cells demonstrated that FGF-10 also promoted cardiomyocyte differentiation.

**Conclusion/Significance:**

FGF-10 induced cardiomyocyte differentiation from ES cells and iPS cells, which may have potential for translation into clinical applications.

## Introduction

Heart failure is a leading cause of morbidity and mortality throughout the world [1], [2]. The dominant cause of heart failure is the loss of myocardium due to coronary artery disease. In the ischemic heart, cardiomyocytes (the contracting heart muscle cells) undergo necrosis, autophagy, and apoptosis, ultimately leading to scar formation. Therefore, strategies to replace damaged cardiomyocytes may prevent heart failure and save lives.

Stem cell therapy is an attractive approach for cardiomyocyte replacement. Recently, mesenchymal stem cells [3], cardiac progenitor cells [4], embryonic stem (ES) cells [5], and induced pluripotent stem (iPS) cells [6] have been reported to be capable of cardiomyocyte differentiation. Amongst them, ES cells and iPS cells may have the best potential. However, the conventional protocol using the hanging drop method to induce cardiac differentiation from ES cells or iPS cells is extremely inefficient [7]–[9], which is a severe obstacle to the use of ES/iPS cells for cardiomyocyte replacement therapy. Accordingly, studies to dissect the molecular pathways mediating ES/iPS cell differentiation into the cardiomyocyte lineage are essential.

The fibroblast growth factor (FGF) family is composed of multifunctional proteins regulating organogenesis, tissue development, and stem cell differentiation. Recent studies have also shown a crucial function of the FGF family during cardiogenesis [10], [11]. For example, the expression of FGF-2 stimulates proepicardial cell differentiation into the epicardial cell lineage, and FGF-10 and FGF receptor (FGFR)-2-III null mice have abnormal heart positioning [12]. Nevertheless, the function of FGFs in ES/iPS cell cardiomyocyte differentiation remains largely unknown. In this study, we used mouse ES cells stably transfected with a cardiac-specific α-myosin heavy chain (αMHC) promoter-driven enhanced green fluorescent protein (EGFP) and mouse iPS cells to investigate cardiomyocyte differentiation. We focused on the expression pattern of FGFs during ES/iPS cell differentiation and carried out experiments to explore the role of FGFs in cardiomyocyte differentiation.

## Results

### Temporal expression profile of FGF members during ES cell cardiomyocyte differentiation

**Figure 1:**
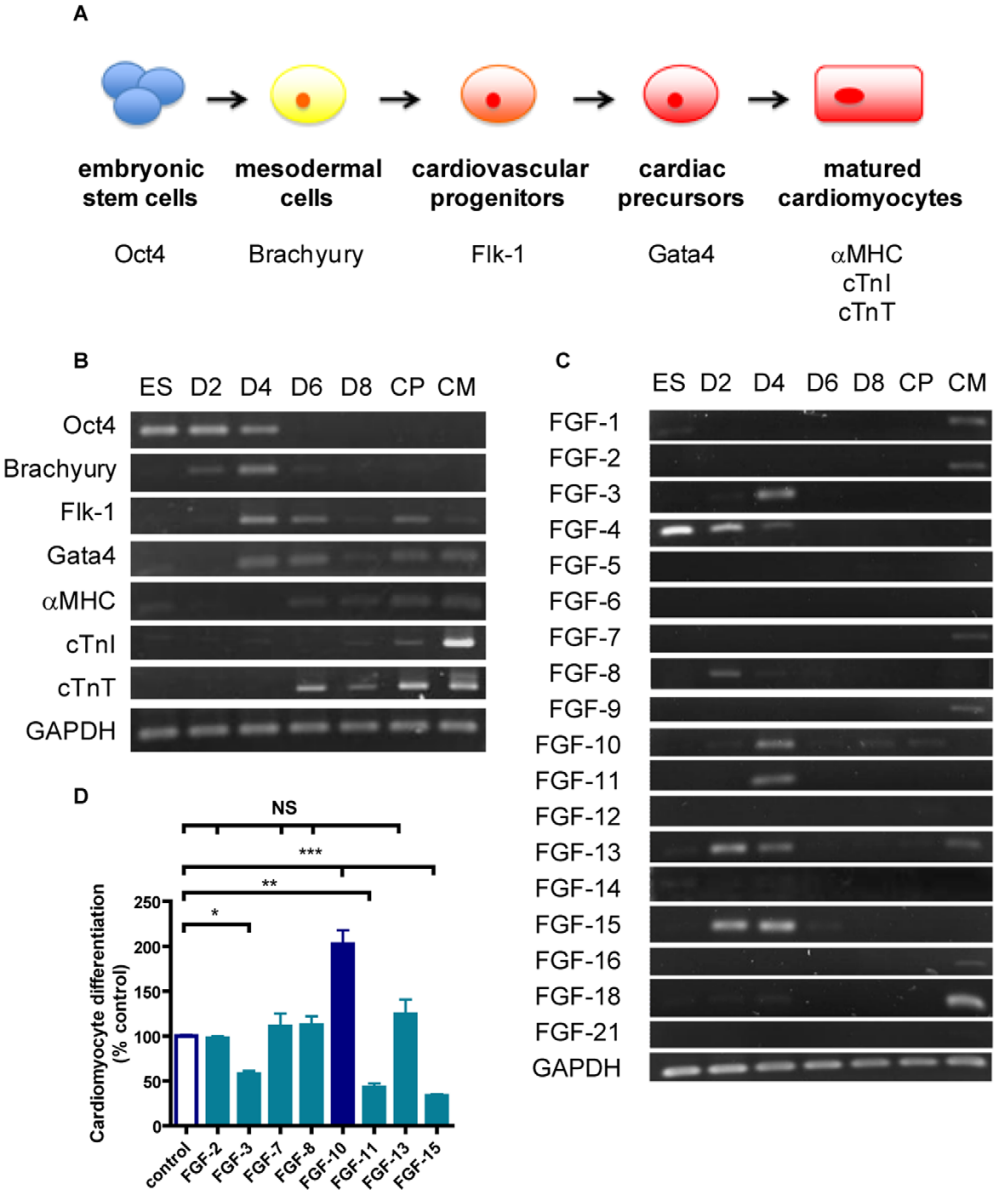
Gene expression profiles of FGF members during cardiomyocyte differentiation from embryonic stem cells. **A**, Schematic presentation of the cardiomyocyte differentiation process showing the intermediate stages denoted by the expression of specific marker genes. **B**, RT-PCR results showing temporal expression patterns of these marker genes on days 2 (D2), 4 (D4), 6 (D6) and 8 (D8) of hanging drop cultivation (n = 3). Purified embryonic stem cell-derived cardiac precursors (CP) and neonatal (1 – 2 days after birth) cardiomyocytes (CM) served as controls. **C**, RT-PCR data showing temporal expression patterns of FGF members (n = 3). Note that FGF-3, FGF-8, FGF-10, FGF-11, FGF-13, and FGF-15 are mainly expressed during the mesodermal and cardiovascular progenitor stages. **D**, Cardiomyocyte differentiation following treatment with FGF-3, FGF-7, FGF-8, FGF-10, FGF-11, FGF-13, and FGF-15 (n = 3). FGF-2 served as a control. ***p<0.001, NS = not significant.

Cardiomyocyte differentiation of ES cells underwent several stages denoted by the temporal expression of specific markers (Figure 1A, and 1B). Undifferentiated ES cells (day 0) highly expressed Oct4, which gradually diminished after differentiation into ectodermal, mesodermal, and endodermal lineages. Mesodermal cells expressed Brachyury from day 2 to day 4, and cardiovascular progenitors, the offspring of mesodermal cells, expressed Flk-1 from day 4 to day 6. Cardiac precursors started to appear on day 4, indicated by the expression of Gata4. Cardiomyocytes, as denoted by the expression of the cardiac structural markers αMHC and cardiac troponin-T, can only be detected by flow cytometry after day 6 of treatment. However, the more mature cardiomyocyte marker, cardiac troponin-I, was not detected until day 8.

One logical approach to increase the proportion of ES cell-derived cardiomyocytes is to take action at an early stage in the differentiation process by augmenting the ratio of ES cells committed to the mesodermal and cardiovascular progenitor lineages. Therefore, members of the FGF family that have a high potential to generate cardiomyocytes may be expressed early in the differentiation process when cells are at the stages of mesodermal and cardiovascular progenitors, that is, from day 2 to day 4. Screening of the temporal expression profile of FGF genes revealed that FGF-3, FGF-8, FGF-10, FGF-11, FGF-13 and FGF-15 were mostly expressed from day 2 to day 4 (Figure 1C). Among them, the exogenous FGF-10 recombinant protein was the most effective in increasing cardiomyocyte differentiation from ES cells by two-fold (Figure 1D).

### FGF-10 is crucial to cardiomyocyte differentiation from ES cells

**Figure 2:**
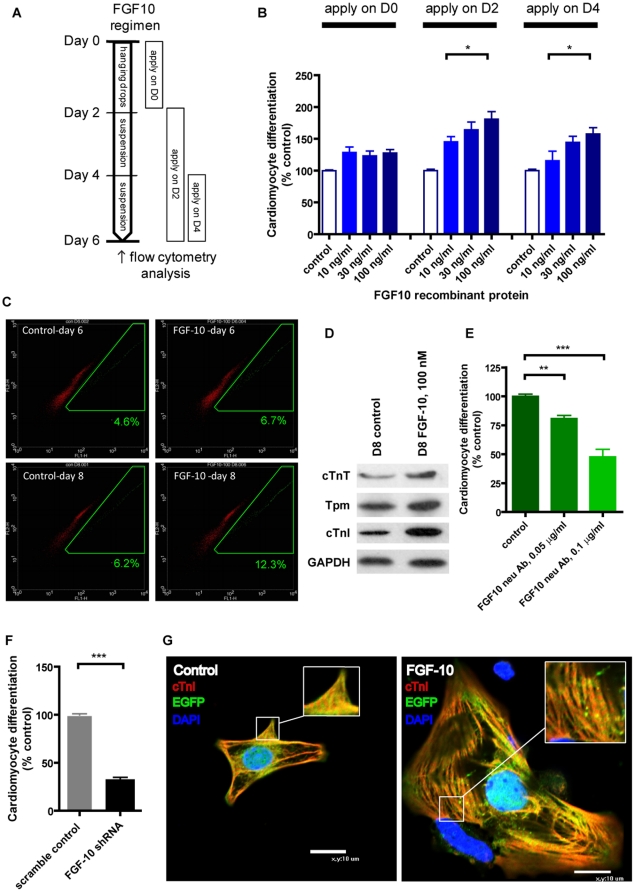
FGF-10 enhances cardiomyocyte differentiation. **A**, Protocol to determine the effect of FGF-10 application. **B**, Cardiomyocyte differentiation of embryonic stem (ES) cells following FGF-10 administration at different times (n = 3). Control, no treatment. **C**, Representative results of flow cytometry for analysis of ES cell differentiation at different time points of FGF-10 treatment. Green boxes indicate EGFP-positive cardiomyocytes (percentages indicated on each panel, n≧3). Control, no treatment. **D**, Immunoblot analysis of mature cardiac markers—cardiac tropomyosin (Tpm), cardiac troponin T (cTnT) and cardiac troponin I (cTnI) in embryoid bodies (n = 3). Control, no treatment. **E**, Differentiation and cell counts of ES cells with FGF-10 neutralizing antibody on day 6 (n = 3). Control, no treatment. *p<0.05, **p<0.01, ***p<0.001. **F**, Cardiomyocyte differentiation with treatment of FGF-10 short hairpin RNA (shRNA, n = 3). Control, cells treated with a scramble shRNA. ***p<0.001. **G**, Confocal imaging of disassociated embryoid bodies using the antibody against cardiac troponin I. Control, no treatment.

Certain growth factors, Wnt-3a being a notable example, are well known to act both stimulatory and inhibitory to cardiomyocyte differentiation depending on the application time during the differentiation process [13]. We found that FGF-10 only enhanced cardiomyocyte differentiation when applied at day 2 or day 4 (Figure 2A, and 2B), which coincided with the expression pattern of the FGF-10 gene (Figure 1C). Also, the stimulatory effect of FGF-10 was concentration-dependent, and immunoblotting confirmed the increased expression of three structural proteins cardiac tropomyosin (Tpm), cardiac troponin-T (cTnT), and cardiac troponin-I (cTnI) (Figure 2B, 2C and 2D). EGFP-positive cells were observed to have more synchronized beating in the FGF-10 treatment group through real-time imaging of the contractile embryoid bodies (Video S1, S2, S3, S4) as well as a more obvious sarcomeric structure through confocal imaging using antibodies against cTnI, cTnT, and Tpm (cTnI data shown in Figure 2G, other data not shown). Our data showed that the cardiomyocyte differentiation ratio increased over time and reached ∼14% in the FGF-10 treated group on day 8 and ∼20% if we prolonged the differentiation time to 14 days (Figure S1A). The group treated with 100 ng/ml of FGF-10 had an optimal cardiomyocyte differentiation ratio that was significantly higher than the group treated with 10 ng/ml of FGF-10 (Figure 2B). Therefore, 100 ng/ml of FGF-10 was used for all subsequent treatments.

Not only does FGF-10 promote cardiomyocyte differentiation, it is crucial during normal cardiomyocyte differentiation process in ES cells. The importance of FGF-10 was demonstrated by the finding that both FGF-10 neutralizing antibody, which inhibited the activation of Akt by FGF-10, and FGF-10 short hairpin RNA (shRNA) blocked cardiomyocyte differentiation without affecting the cell number (Figure 2E, 2F and Figure S2). Moreover, FGF-10 did not influence the ES cell cycle (data not shown), indicating that the FGF-10 response was specific to cardiomyocyte differentiation and not a consequence of ES cell proliferation increase or ES cell apoptosis decrease.

To gain more insight on the effect of FGF-10 in the cardiomyocyte differentiation process, we examined the expression pattern of several markers using cells treated with FGF-10 recombinant protein and shRNA for knockdown (Figure S3). From the semi-quantitative PCR results, FGF-10 increased the expression of Brachyury, Flk-1 and mature cardiac markers αMHC, cTnI and cTnT. Interestingly, FGF-10 knockdown resulted in downregulation of Flk1, Gata4, αMHC, cTnT, and cTnI, but not Brachyury. Together, these results demonstrate that FGF-10 may induce the mesodermal and cardiogenic gene expression. However, the increased mesoderm differentiation by FGF-10 is sufficient but may not be required for cardiac differentiation. In addition to mesoderm induction, FGF-10 also promoted expression of the ectoderm genes at early stages (day 3 to day 5) and that of the endoderm genes in later differentiation process (day 5 to day 7) (Figure S1B, S1C, and S1D)

### Response to FGF-10 is mediated via FGF receptors

**Figure 3:**
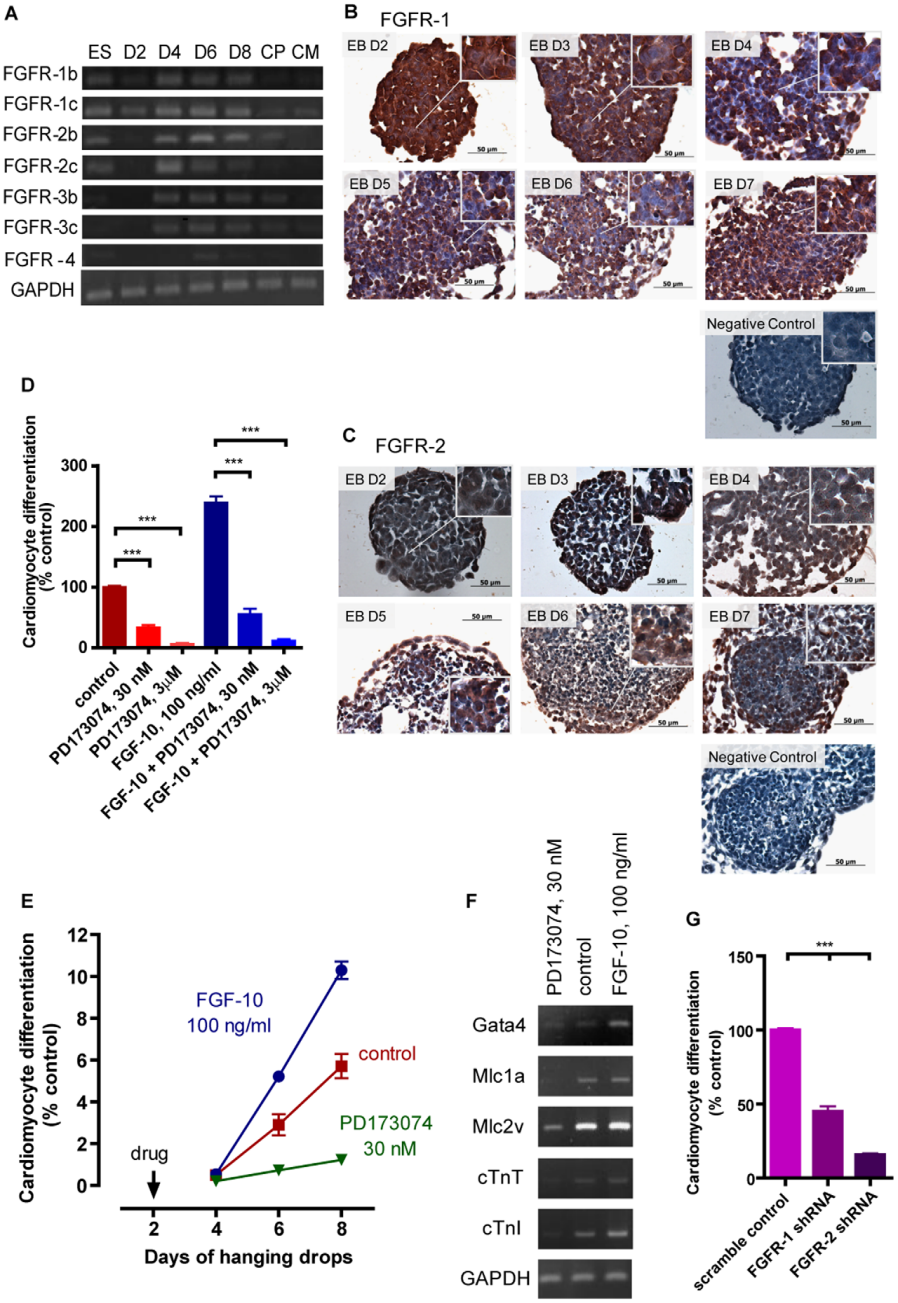
The FGF-10 response is mediated via FGF receptors. **A**, RT-PCR results showing gene expression of all 7 isoforms of FGF receptors (FGFRs) during embryonic stem cell cardiomyocyte differentiation (n = 3). **B-C**, Photomicrographs of embryonic bodies (EBs) stained for FGFR-1 (B) and FGFR-2 (C) at the indicated days. Control, without first antibody. **D**, Cardiomyocyte differentiation in the presence of the specific small molecule inhibitor of FGFR, PD173074, alone and along with FGF-10. (n = 3). **E**, Cardiomyocyte differentiation in response to FGF-10 and PD173074 at the indicted times in hanging drop culture. **F**, RT-PCR data for the expression of cardiomyocyte-specific genes following treatment with FGF-10 or FGFR inhibitor. Control, GAPDH. **G**, Cardiomyocyte differentiation in the treatment of FGFR-1, FGFR-2 short hairpin RNA (shRNA, n = 3). Control, cells treated with a scramble shRNA. *p<0.05, ***p<0.001.

Members of the FGF family, with the exception of FGF-11, -12, -13 and -14, exert their functions via FGF receptors (FGFRs) [14]. Genes encoding all seven isoforms of FGFR RNA, as well as FGFR-1 and FGFR-2 proteins were expressed during the cardiomyocyte differentiation process of ES cells (Figure 3A, 3B and 3C). The small molecule FGFR-specific inhibitor, PD173074, diminished the degree of cardiomyocyte differentiation with or without FGF-10 stimulation, and without altering the cell number (Figure 3D, 3E and data not shown). FGFR inhibition also downregulated the expression of the cardiomyocyte-specific genes Gata4, Mlc1a, Mlc2v, cTnT and cTnI (Figure 3F). FGF-10 is known to primarily activate FGFR-2 with little stimulatory action on FGFR-1 [15]. To determine which FGFR isoforms contribute to the FGF-10-mediated cardiomyocyte differentiation response, shRNA interference was used. FGFR-1 and FGFR-2 shRNA reduced cardiomyocyte differentiation from both non-stimulated and FGF-10-stimulated ES cells, while the scramble control vector had no effect (Figure 3G). Reduced cardiomyocyte differentiation was more obvious in the FGFR-2 knockdown group than that in FGFR-1 knockdown. Therefore, FGFR-2 may play a more important role in FGF-10 promoted cardiomyocyte differentiation from ES cells. To prove that the increased percentage of cardiomyocyte differentiation was specifically due to FGFR-2 activation by FGF-10 rather than by other FGFR-2 activators, ES cells were treated with FGF-7 and evaluation of cardiomyocyte differentiation showed no significant difference from that of the control (Figure 1D).

### The enhancement of cardiomyocyte differentiation by FGF-10 is also apparent in iPS cells

Research on iPS cells is gaining momentum because of their accessibility, similar pluripotency, and fewer ethical concerns than ES cells [16], [17]. While iPS cells have been shown to differentiate into cardiomyocytes for cardiac repair, the cardiomyocyte differentiation ratio is as low as that of ES cells [18]. It is thus instructive to examine the effects of FGF-10 on cardiomyocyte differentiation in iPS cells.

**Figure 4:**
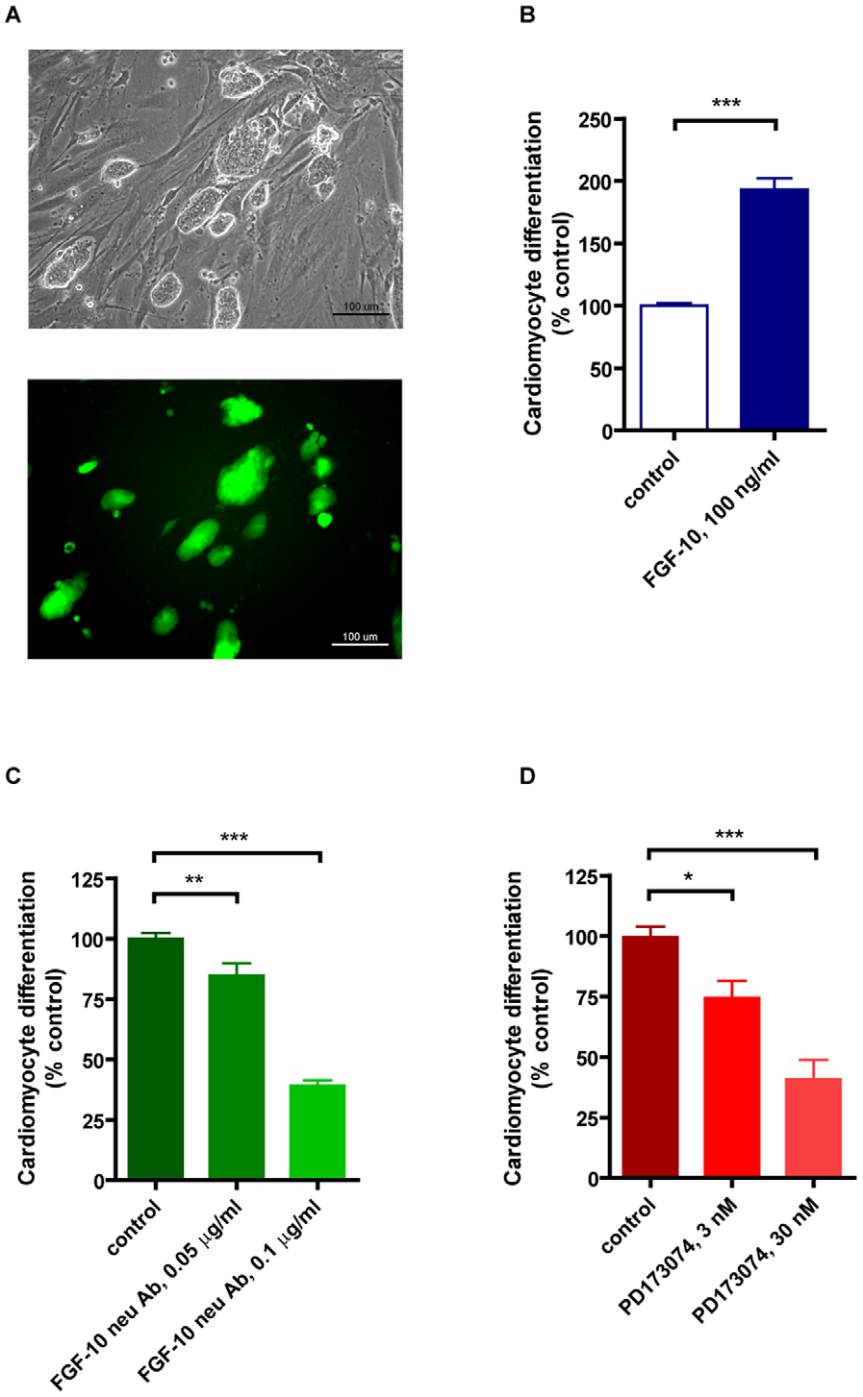
FGF-10 also augments cardiomyocyte differentiation from induced pluripotent stem cells. **A**, The induced pluripotent stem (iPS) cell line (Nanog promoter-driven EGFP) used in the present study. **B–D**, Results of cardiomyocyte differentiation from iPS cells in the treatment of FGF-10 (B), FGF-10 neutralizing antibody (C) or FGFR inhibitor PD173074 (D). Control, no treatment. *p<0.05, **p<0.01, ***p<0.001.

Similar to its effect on ES cells, FGF-10 doubled cardiomyocyte differentiation from iPS cells (Figure 4A, and 4B), while FGF-10 inhibition by its neutralizing antibody and FGFR inhibition by the inhibitor PD173074 reduced iPS cell cardiomyocyte differentiation (Figure 4C, and 4D). Therefore, the FGF-10-FGFR axis is essential to activate iPS cells into the cardiomyocyte lineage, and FGF-10 is a promising candidate in augmenting this process.

### FGF-10 modulates the expression of genes that regulate cardiogenesis

**Figure 5:**
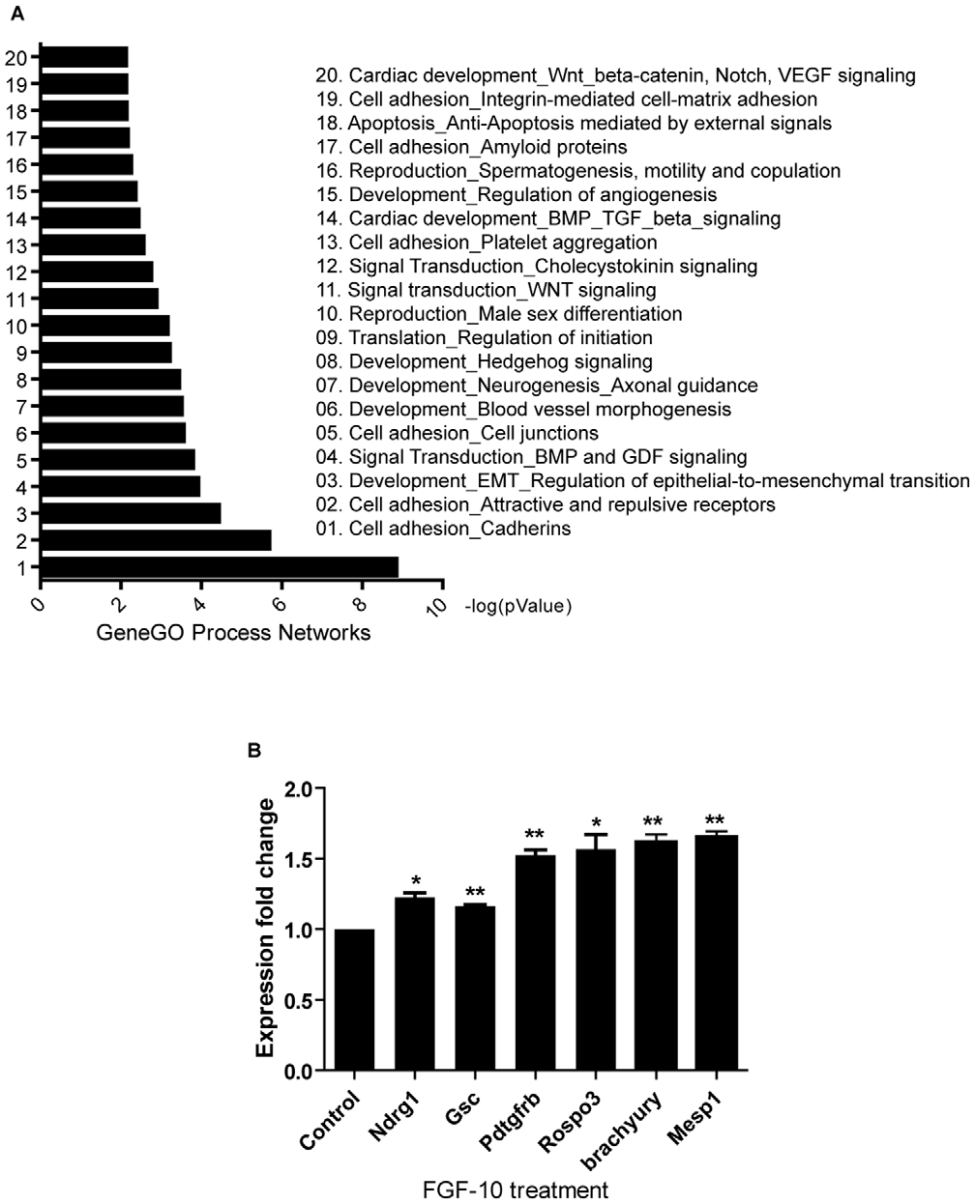
FGF-10 modulates the expression of cardiac related genes and pathways. **A**, Gene ontology process network analysis showing predicted functions of FGF-10 mediated upregulated genes. (p-value ≦0.01). **B**, Gene validation using quantitative real-time PCR, *p<0.05, **p<0.01, ***p<0.001.

FGF-10 significantly upregulated cardiomyocyte differentiation on day 2 rather than day 4 when FGF-10 was highly expressed (Figure 2B). We anticipated the effect of FGF-10 on cardiomyocyte differentiation is to activate differentiation of mesodermal lineages. To shed light on how FGF-10 governs the course of cardiomyocyte differentiation, the global gene expression was profiled by Affymetrix Mouse Genome 430 2.0 Array. As predicted, the mesodermal markers Brachyury, Goosecoid, and surprisingly, the early cardiac marker, Mesp1 were upregulated upon FGF-10 treatment (Figure 5B and Table S1). Results from Gene Ontology analysis suggested that the genes involved in cardiac developmental pathways were induced by FGF-10. In addition, the effect of FGF-10 was not limited to the cardiac lineage. Gene Ontology also revealed the role of FGF-10 in cell adhesion and tissue development such as in neurogenesis, blood vessel morphogenesis, and spermatogenesis (Figure 5A).

### FGF-10 enhances cardiomyocyte differentiation from ES cells *in vivo*

**Figure 6:**
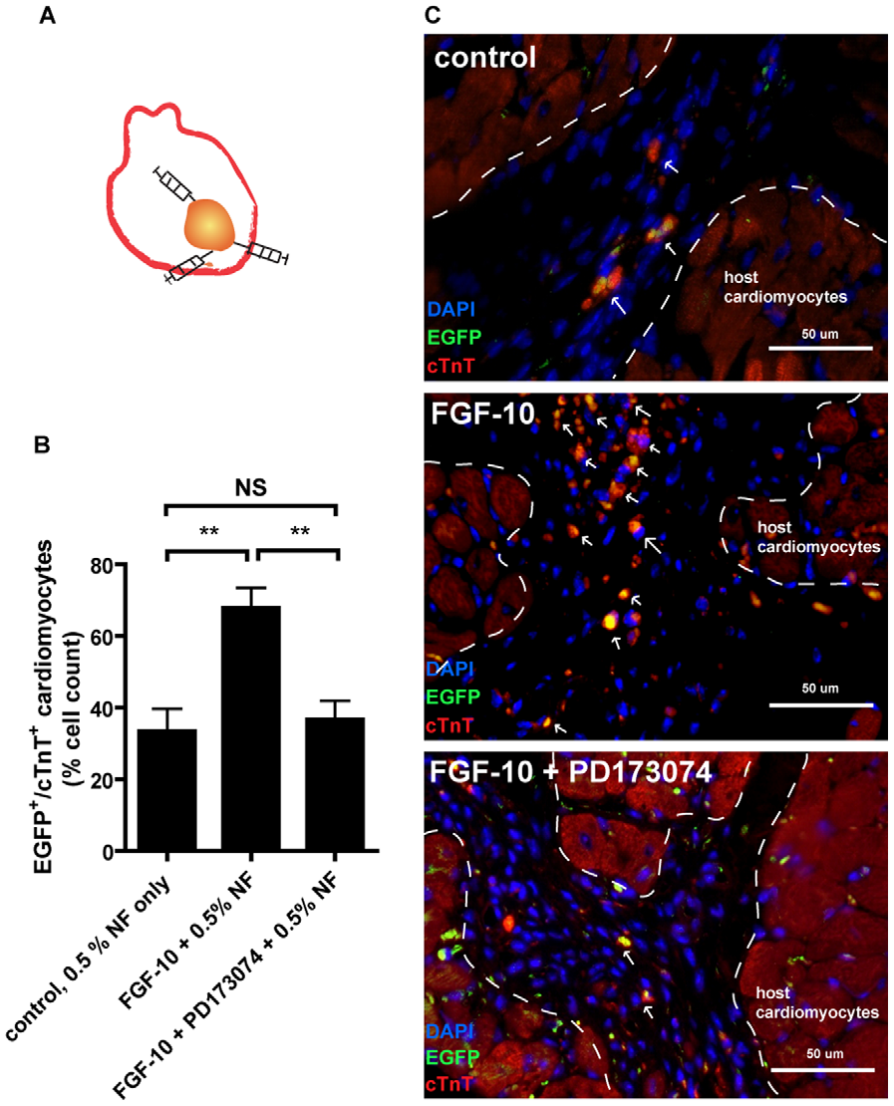
FGF-10 promotes cardiomyocyte differentiation from embryonic stem cells *in vivo*. **A**, Schematic illustration of the intramyocardial administration of embryonic stem (ES) cells and peptide nanofibers (NF). **B**, Statistical summary of EGFP-cTnT double positive cells per total cell count from images such as in C (n = 4; **p<0.01). **C**, Photomicrographs of ES cells from hearts injected with cells and NF only (upper panel), along with FGF-10 (middle panel) and with FGF-10 plus PD173074 (lower panel). Also shown are cardiomyocytes stained for EGFP (green), cardiac Troponin-T (cTnT, red) and DAPI (blue). Note the co-localization of EGFP and cTnT (yellow).

As a proof of concept, the cardiomyocyte differentiation response to FGF-10 was examined in an *in vivo* setting (Figure 6A). Self-assembling peptide nanofiber hydrogels were used to form a defined intramyocardial microenvironment where FGF-10 and embryoid bodies (EBs) were injected into [19]. FGF-10 significantly enhanced EGFP-cTnT double positive cells per total cell count at the injection area (Figure 6B and 6C). The number of the double positive EGFP-cTnT cells was decreased in the FGF-10 mixed with PD173074 compound group. This indicates that FGF-10 can promote cell differentiation into cardiomyocytes which expressed late stage cardiac markers such as cTnT though clear sarcomeric structure cannot be observed, possibly due to the limitation of cell spreading in the injected hydrogel microenvironment. Therefore, FGF-10 increases cardiomyocyte differentiation of ES cells *in vivo*.

## Discussion

The major challenge of using ES cell-based cardiomyocyte replacement therapy is the inefficiency of the conventional protocol to generate cardiomyocytes [8], [9]. In the present study, we identified FGF-10 as a key factor in ES/iPS cell differentiation into cardiomyocytes. FGF-10 doubled the proportion of ES/iPS cells entering the cardiomyocyte lineage. The FGFR-1 and FGFR-2 were required for the FGF-10 effect. These findings suggest that FGF-10 may serve as a target in developing novel therapies for cardiac regeneration.

To explore the molecular mechanisms controlling ES cell differentiation into cardiomyocytes, we used an αMHC-based reporter system to profile the temporal gene expression of cardiac markers during ES cell differentiation. Previously, other groups used the Nkx2.5 reporter system to investigate stem cell differentiation into cardiomyocytes [20]–[22]. Because Nkx2.5 is an earlier marker or cardiac precursor cells than αMHC [20], a reporter system using Nkx2.5 may not be as representative as αMHC. Cells expressing Nkx2.5 may differentiate into lineages other than cardiomyocytes, such as conduction system cells, endothelial cells, and vascular smooth muscle cells [21], [22].

We focused on the FGF family members and identified the expression pattern of several FGFs at the mesodermal and cardiovascular progenitor stages, around two to three days after EB formation. Among the candidates, only FGF-10 consistently enhanced ES cell and iPS cell differentiation into cardiomyocytes. Interestingly, the time of FGF-10 addition was critical for cardiac induction; there was no effect on cardiomyocyte differentiation when we applied FGF-10 at day 0, but there was a ∼2-fold increase of cardiomyocyte differentiation when applying FGF-10 at day 2 or day 4. This suggests that FGF-10 interacts with mesodermal cells and commits them into precursors of the cardiomyocyte lineage. While we showed that FGF-10 may affect the cardiomyocyte lineage, its effect on other cardiovascular lineages is unclear and requires further investigation. Because of the low efficiency of ES cell-derived cardiomyocytes, the difficulty of purification is another obstacle that must be overcome.

FGF signaling is triggered through FGFR activation [14]. In our study, FGFR-1 and FGFR-2 were expressed during ES cell cardiomyocyte differentiation. PD173074, an inhibitor of FGFRs, and FGFR-1, FGFR-2 shRNA reduced ES cell cardiomyocyte differentiation, thus demonstrating that the FGF-FGFR signaling pathway is critical in the control of ES/iPS cell differentiation into cardiomyocytes [12], [23]. Reduced cardiomyocyte differentiation was much more significant in the FGFR-2 knockdown group than that in FGFR-1 knockdown. Thus, our findings suggest that FGFR-2 may be a potential biomarker to identify cells committed to cardiac lineages. Therefore, in the future we may pursue a genetic fate-mapping approach to establish that FGF-10-FGFR-2 signaling plays a pivotal role in ES/iPS cell cardiac differentiation.

iPS cells are known to differentiate into cardiomyocytes [17], [24], [25]. The main advantages of iPS cells are the absence of an immune response and minimal ethical concerns, making these cells a powerful tool for stem cell therapy. Unfortunately, iPS cell cardiomyocyte differentiation ratio is extremely inefficient for therapies, much like ES cells [26]. Therefore, we also set out to test whether FGF-10 augments the ratio of iPS cell differentiation into cardiomyocytes. Interestingly, our results showed that FGF-10 and its inhibition had similar effects on iPS cells and ES cells. Our data revealed that the function of FGF-10 in regulating stem cell cardiac differentiation is similar in both ES cells and iPS cells.

We performed microarray in search for a possible mechanism of how FGF-10 promotes cardiomyocyte differentiation from ES cells. The enhancement of FGF-10 on cardiomyocyte differentiation applied on day 2 better than on day 4 when the endogenous FGF-10 highly expressed indicated the possibilities of direct or indirect interaction of regulators involved in mesodermal lineages. Array data revealed mesodermal markers such as Brachyury, Goosecoid, Rspo3, and early cardiac marker Mesp1 [27]–[30] were upregulated following FGF-10 treatment. Gene ontology analysis also indicated that FGF-10 may act through BMP, TGF-β, Wnt-β-catenin, Notch, and VEGF signaling to induce cardiomyocyte differentiation, although the specific mechanisms have yet to be determined. FGF-10 not only promoted cardiomyocyte differentiation via activating mesodermal lineages at early stage of differentiation but also increased the expression of cTnT, and cTnI, two mature cardiac markers, in ES cell-derived cardiomyocytes at later differentiation stage.

To explore the *in vivo* role of FGF-10 in cardiomyocyte differentiation, we injected FGF-10 incubated with peptide nanofibers into the myocardium. Interestingly, similar to *in vitro* results, FGF-10 increased the ratio of EGFP-positive cells. However, while these EGFP-positive cells co-expressed cTnT, the indication that these cells turned into mature cardiomyocytes is yet to be confirmed because the sarcomeric structure is not obvious. Our present two-week study falls short in answering some pending concerns, such as teratoma formation and immune response to FGF-10-stimulated ES cell-based therapy. A long term *in vivo* study is required to verify the roles of FGF-10-guided ES cell differentiation in aiding cardiac repair after myocardial infarction.

In conclusion, we discover that FGF-10 is a novel regulator of cardiomyocyte differentiation from ES cells and iPS cells. Our results not only help in understanding the molecular mechanisms mediating ES/iPS cell cardiac differentiation, but also have the potential for translation into clinical applications.

## Materials and Methods

### Ethics Statement

All animal research procedures were approved by the National Cheng Kung University Animal Care and Use Committee. The investigation conformed to the *Guide for the Care and Use of Laboratory Animals* published by the US National Institutes of Health (NIH Publication No. 85-23, revised 1996).

### Culture of mouse ES-αMHC-EGFP cells and cardiomyocyte differentiation induction

Mouse ES-αMHC-EGFP cells were obtained by transfection of the α-myosin heavy chain (MHC) promoter-driven enhanced green fluorescent protein (EGFP) reporter into the CGR8 ES cell line [8]. For normal maintenance, ES cells were cultured in Glasgow Minimum Essential Medium (Sigma, St. Louis, MO) supplemented with 15% knockout serum replacement (Invitrogen, Carlsbad, CA), 1% non-essential amino acids (Invitrogen), 1 mM sodium pyruvate (Invitrogen), 1% penicillin/streptomycin (Invitrogen), 0.1 mM -mercaptoethanol (Sigma) and 1000 U/mL leukemia inhibitory factor (Chemicon, Temecula, CA). To initiate cardiomyocyte differentiation (day 0), ES cells were cultured in hanging drops of 400 cells per 20 *µ*L of differentiation medium (composition identical to maintenance medium except without leukemia inhibitory factor and 10% fetal bovine serum replacing knockout serum replacement) to form EBs. On day two, EBs were transferred into fresh differentiation medium for suspension cultivation in non-adhering petri dishes. To screen the effects of FGF members on cardiomyocyte differentiation, FGF-2, FGF-3, FGF-10 (R&D Systems, Minneapolis, MN), FGF-11, FGF-13 (both from Abnova, Taipei, Taiwan) and FGF-7, FGF-8, FGF-15 (all from PeproTech, Rocky Hill, NJ) recombinant proteins were applied on day 2 at 100 ng/ml.

### Culture of mouse iPS cells

Mouse iPS-MEF-Ng-20D-17 cells were obtained from the Cell Bank, Riken BioResource Center (Tsukuba, Japan). These cells contained a Nanog promoter-driven EGFP reporter [31]. Mouse iPS cells were cultured with or without mouse embryonic fibroblasts as a feeder layer. The procedures of maintenance and cardiomyocyte differentiation initiation of iPS cells were otherwise identical to ES cells as described above.

### Temporal gene expression profiling

RNA was extracted from ES cells, EBs at various time points (day 2, day 4, day 6 and day 8), ES cell-derived cardiac precursors and neonatal mouse cardiomyocytes using Trizol reagent (Invitrogen Carlsbad, CA) according to the manufacturer's protocol. Semi-quantitative reverse transcription-polymerase chain reaction (RT-PCR) was subsequently performed. The primer sequences used for RT-PCR were as follows:

GAPDH, forward: 5′-AACGACCCCTTCATTGAC-3′, reverse:

5′-TCCACGACATACTCAGCAC-3′;

Oct4, forward: 5-′CACGAGTGGAAAGCAACTCA-3′, reverse: 5′AGATGGTGGTCTGGCTGAAC-3′;

Brachyury, forward: 5′-GCTCATCGGAACAGCTCTCCAACC-3′, reverse:

5′-GGAGAACCAGAAGACGAGGACGTG-3′;

Flk-1, forward: 5′-GCTTTCGGTAGTGGGATGAA-3′, reverse:

5′-GGAATCCATAGGCGAGATCA-3′;

Gata4, forward: 5′-GCAGCAGCAGTGAAGAGATG-3′, reverse:

5′-GCGATGTCTGAGTGACAGGA-3′;

αMHC, forward: 5′-GAGGACCAGGCCAATGAGTA-3′, reverse:

5′-GCTGGGTGTAGGAGAGCTTG-3′;

Cardiac troponin I (cTnI), forward: 5′-CTCCTCTGCCAACTACCGAG-3′, reverse:

5′-CTCAAACTTTTTCTTGCGGC-3′;

Cardiac troponin T (cTnT), forward: 5′-ATCCCCGATGGAGAGAGAGT-3′, reverse:

5′-TTCCCACGAGTTTTGGAGAC-3′;

Mlc1a, forward: 5′-CCTCAAGGACTCTGCCTTTG-3′, reverse:

5′-TCTTCTCTCCCAGGGTAGCA-3′;

Mlc2v, forward: 5′-AGCCTTCACAATCATGGACC-3′, reverse:

5′-CCTCCCTGCTTGTGTGGTCA-3′;

FGF-1, forward: 5′-GGTTCAACCTGCCTCTAGGA-3′, reverse:

5′-ATAAAAGCCCTTCGGTGTCC-3′;

FGF-2, forward: 5′-TATCAAGGGAGTGTGTGCCA-3′, reverse:

5′-TATGGCCTTCTGTCCAGGTC-3′;

FGF-3, forward: 5′-CTTCGGATCACTACAACGCA-3′, reverse:

5′-GGGCAGGAAGAGAGAGGACT-3′;

FGF-4, forward: 5′-CGAGGGACAGTCTTCTGGAG-3′, reverse:

5′-GACACTCGGTTCCCCTTCTT-3′;

FGF-5, forward: 5′-GTCTTCTGCCTCCTCACCAG-3′, reverse:

5′-GAAGTGGGTGGAGACGTGTT-3′;

FGF-6, forward: 5′-AATTGGGAAAGCGGCTATTT-3′, reverse:

5′-CCCGTCCATATTTGCTCAGT-3′;

FGF-7, forward: 5′-CAAACGGCTACGAGTGTGAA-3′, reverse:

5′-TAAGGCAACGAACATTTCCC-3′;

FGF-8, forward: 5′-TTGCACTTGCTGGTTCTCTG -3′, reverse:

5′- ACTCGGACTCTGCTTCCAAA -3′;

FGF-9, forward: 5′-TTCCCCAACGGTACTATCCA-3′, reverse:

5′-CTTTGTCAGGGTCCACTGGT-3′;

FGF-10, forward: 5′-TTTTTGGTGTCTTCGTTCCC-3′, reverse:

5′-CTGACCTTGCCGTTCTTCTC-3′;

FGF-11, forward: 5′-GTCGCTTTAAGGAGTGCGTC-3′, reverse:

5′-CACTGTGGAGAGAAGGCTCC-3′;

FGF-12, forward: 5′-TTCCTGTAGGACTGCGTGTG -3′, reverse:

5′-TTGAGCGTCCTTGCTTTTCT -3′;

FGF-13, forward: 5′-GAAATCCAATGCCTGCAAGT-3′, reverse:

5′-AGGTGTGAAATGTTCCGAGG-3′;

FGF-14, forward: 5′-AGAGCCTGGTTTTTGGGATT-3′, reverse:

5′-GTTGACTGGTTTGCCTCCAT-3′;

FGF-15, forward: 5′-GCTGGTCCCTATGTCTCCAA-3′, reverse:

5′-TGGTCCTGGAGCTGTTCTCT-3′;

FGF-16, forward: 5′-GTCTTTGCCTCCTTGGACTG-3′, reverse:

5′-TCTCTCCGAGTCCGAGTGTT-3′;

FGF-17, forward: 5′- TCAAACACAGGGGGAGAATC-3′, reverse:

5′- TCTGCTGCCGAATGTATCTG -3′;

FGF-18, forward: 5′-GTGCTTCCAGGTTCAGGTGT-3′, reverse:

5′-AGCCCACATACCAACCAGAG-3′;

FGF-21, forward: 5′- CTGGGGGTCTACCAAGCATA -3′, reverse:

5′- CAGGATCAAAGTGAGGCGAT -3′

FGFR-1b, forward: 5′-TTCAGTGGCTGAAGCACATC-3′, reverse:

5′-CATGCAGAGTGATGGGAGAG-3′;

FGFR-1c, forward: 5′-TTCAGTGGCTGAAGCACATC-3′, reverse:

5′-GGAAGTCGCTCTTCTTGGTG-3′;

FGFR-2b, forward: 5′-CTGCCTGGTGGAGAATGAAT-3′, reverse:

5′-TCACATTGAACAGAGCCAGC-3′;

FGFR-2c, forward: 5′-GGAGGGGATGTGGAGTTTGT-3′, reverse:

5′-CAGAACTGTCAACCATGCA-3′;

FGFR-3b, forward: 5′-GGAGTTCCACTGCAAGGTGT-3′, reverse:

5′-ACGCAGAGTGATGGGAAAAC-3′;

FGFR-3c, forward: 5′-GGAGTTCCACTGCAAGGTGT-3′, reverse:

5′-CCAGCCTCATCAGTTTCCAT-3′;

FGFR-4, forward: 5′-CGTGGTCGTCACTGGTACAA-3′, reverse:

5′-TGATGGAGGTTAAGGAGTC-3′.

### Quantitative real-time PCR

RNA of treated and non-treated EBs were extracted on day 3 using Trizol reagent (Invitrogen) according to the manufacturer's protocol. RNA was analyzed with quantitative real-time PCR (qPCR). Melting curve analyses and PCR product sequencing were performed to verify primer specificities. qRT-PCR was repeated at least three times using the following conditions. Each of the reaction mixtures contained 10 µl of SYBR Green master mix (Applied Biosystems), 5 pmoles each of forward and reverse primers and 5 µl of 100 times diluted cDNA. The primer sequences used for qPCR were as follows:

ndrg1, forward: 5′-AGTGCACACGTACCGCCAGC-3′, reverse:

5′-ACTGGTGTGCGAGCGGCTTC-3′;

gsc, forward: 5′ -CCAGCAGTGCTCCTGCGTCC-3′, reverse:

5′-CCCTCTTCTCCGGCGAGGCT-3′;

pdgfrb, forward: 5′-CACCTTCTCCAGTGTGCTGA-3′, reverse:

5′-GGAGTCCATAGGGAGGAAGC-3′;

rospo3, forward: 5′ -GCTGCCAAGGAGGCTGTGCA-3′, reverse:

5′-CGGACCCGTGTTTCAGTCCCC-3′;

mesp1, forward: 5′ -TGTACGCAGAAACAGCATCC-3′, reverse:

5′- TTGTCCCCTCCACTCTTCAG-3′;

nestin, forward: 5′ - GCTGGCTGTGGAAGCCCTGG -3′, reverse:

5′- CTGGGCACTGTGGCCTCAGC -3′;

gata3, forward: 5′- CCTGAGCAGCCACCACACCG -3′, reverse:

5′- GCAGGCTGCTGGGTGGGAAG -3′;

sox17, forward: 5′ - TCGACGGCTACCCTCTGCCC -3′, reverse:

5′- CGTGCGGTCCACCTCCCCTA -3′;

transferrin, forward: 5′ - TAAGCTGTCGGAGCCCCGCA -3′, reverse:

5′- AAAGGCCACATCCCCACCGC -3′;

### Cardiomyocyte differentiation quantification by flow cytometry analysis

Cardiomyocyte differentiation from ES cells or iPS cells was quantified by flow cytometry (BD FACSCalibur, BD Bioscience, Bedford, MA) of cardiac specific markers (aMHC promoter-driven EGFP or cTnT). EBs were collected on day 6 (or as specified otherwise) and dispersed into single cells by trypsin (Invitrogen). Cells of ES cell origin were subsequently examined for EGFP signals. For cells of iPS cell origin, they were first stained with anti- cTnT antibody (1∶100, clone CT3; Developmental Studies Hybridoma Bank, Iowa City, IA) and Alexa Fluor 568 secondary antibody (1∶1000; Invitrogen) before flow cytometry analysis. Cardiomyocyte differentiation was quantified by the number of EGFP positive cells (ES cell-derived cardiomyocytes) or cTnT positive cells (iPS cell-derived cardiomyocytes). A total of 10000 cells were analyzed at a time and the assay was repeated at least three times to average the percentage of cardiomyocyte differentiation.

### FGF-10 neutralization and FGFR inhibition

For loss-of-function studies, day 2 EBs were cultivated with FGF-10 neutralizing antibody (BioVision, Mountain View, CA) or a small-molecule FGFR inhibitor, PD173074 (Sigma, St. Louis, MO).

### Gene knockdown assay

Bacteria transformed with FGF-10, FGFR-1, FGFR-2 shRNA in pLKO.1-puro lentiviral backbone were obtained from National RNAi Core Facility (Academia Sinica, Taipei, Taiwan) and scramble control vector was obtained from Addgene. Further culture and expansion were conducted according to the manufacturer's protocol. Purified FGF-10, FGFR-1, FGFR-2 shRNA plasmid was then transfected into 293T cells with Lipofectamine (Invitrogen) together with an envelope expression plasmid (pMD2.G) and a packaging vector (psPAX2) to generate the shRNA-containing lentivirus. Each lentivirus was then infected into ES cells according to the National RNAi Core Facility protocol.

### Immunohistochemical staining

EBs collected on day 2 to day 7 were fixed, paraffin-embedded, and subjected to immunohistochemical staining with anti-FGFR-1 antibody (1∶100, clone C-15; Santa Cruz Biotechnology, Santa Cruz, CA) or anti-FGFR-2 antibody (1∶100, clone 133730; R&D Systems), and then with 3,3′-diaminobenzidine (DAB) for visualization (Vector Laboratories, Burlingame, CA).

### Cell cycle analysis

ES cells were treated with FGF-10 for 24 h, fixed and subjected to propidium iodide (1 mg/ml; Sigma) staining and flow cytometry analysis as previously described [9].

### Preparation of a EBs-peptide nanofiber mixture for transplantation

A mixture of EBs and peptide nanofibers was prepared by suspending twenty ES cells-derived day 2 EBs into 20 *µ*l of 0.5% peptide nanofiber (RAD16-II peptide: AcN-RARADADARARADADA-CNH_2_, Synpep, Dublin, CA) in phosphate buffered saline (pH 7.4). FGF-10 (100 ng/ml) or 30 nM PD173074 was added into the mixture as needed.

### In vivo evaluation of FGF-10 on cardiomyocyte differentiation

C57BL/6 mice (20–25 g, acquired from the National Laboratory Center, Taipei, Taiwan) were anesthetized with Zoletil (50 mg/kg, Virbac, France) and Rompun (0.2 ml/kg, Bayer Healthcare, Germany), artificially ventilated, and had their hearts exposed. Three groups: 0.5% nanofibers only, 0.5% nanofibers +100 ng/ml FGF-10, 0.5% nanofibers +100 ng/ml FGF-10+30 nM PD173074, mixed with day 2 EBs were subsequently injected into the free wall of left ventricle myocardium by three injections (n = 4, in each group) [32]. The chest was then closed, and the mice were allowed to recover. Two week later, mice were sacrificed, and the hearts were harvested, paraformaldehyde-fixed and paraffin-embedded. Immunohistochemistry was subsequently performed using antibodies against EGFP (1∶100, clone 1E4; MBL, Woburn, MA), cTnT (1∶100) and then incubated with Alexa Fluor-conjugated secondary antibodies (1∶200, Molecular probes, Invitrogen). After counterstaining with DAPI (Sigma-Aldrich), sections were mounted and observed under a fluorescence microscope. Six sections per heart were evaluated for ES cell incorporation and EGFP-cTnT double positive cells were counted and divided by the total cell count.

### Microarray analysis

Two-day EBs were treated with or without 100 ng/ml FGF10 and RNA was obtained 24 hours later for microarray analysis, which were performed by the Affymetrix Gene Expression Service Lab (http://ipmb.sinica.edu.tw/affy/), supported by the Academia Sinica. The full normalized data are in Supplementary Data 1. A total of 172 upregulated genes (FGF-10 treatment/without treatment, p-value <0.001) were analyzed in MetaCoreTM of GeneGo Inc. for enrichment of gene ontology terms. Terms with *P* values adjusted for multiple testing ≤ 0.01 were considered enriched. Microarray report conformed to the MIAME guidelines, and the data was submitted to Gene Expression Omnibus.

### Statistical analysis

Results were expressed as mean ± S.E.M. for at least three independent experiments. Data were analyzed by Student t-test or one-way or two-way analysis of variance with Newman-Keuls post-hoc test where appropriate. Statistical significance was set as p<0.05 (two-tailed).

## Supporting Information

Table S1 Microarray results of gene symbol, accession number, fold change and predicted functions of the up- and down- regulated genes in ES cells treated with FGF-10.
(0.47 MB XLS)

Figure S1 The effect of FGF-10 in controlling ES cell differentiation. A, The percentage of cardiomyocyte differentiation of ES cell treated with FGF-10 over time from day 7 to day 14. B, the time course study of the three germ layer markers upon FGF-10 treatment with quantitative real-time PCR.
(6.68 MB TIF)

Figure S2 The study of ES cell toxicity under the treatment of FGF receptor inhibitor PD173074. A, Immunoblot analysis of phospho- and total-Akt in embryoid bodies of different treatment. FGF-10, 100 ng/ml. Neutralizing antibody, 0.1μg/ml. PD173074, 30 nM. Control, no treatment. B-D, Total cell count of ES cells treated with an FGF-10 neutralizing antibody, the FGFR inhibitor PD173074, or a lentivirus contained FGF -10, FGFR-1 or FGFR-2 shRNA. Control, no treatment.
(4.26 MB TIF)

Figure S3 Gene expression profiles of ES cells treated with FGF-10 protein or shRNA. A, RT-PCR results showing the temporal expression pattern of Brachyury, Flk1, Gata4, αMHC, cTnI and cTnT on day 3 (D3), 5 (D5), 7 (D7) and 9 (D9) during hanging drop cultivation of ES cells after the treatment of 100 ng/ml FGF-10. (n≧3, *p < 0.05, **p < 0.01, ***p < 0.001.). B, RT-PCR results showing the temporal expression pattern of Brachyury, Flk1, Gata4, αMHC, cTnI and cTnT on day 3 (D3), 5 (D5), 7 (D7) and 9 (D9) during hanging drop cultivation of ES cells after FGF-10 knockdown by shRNA (n≧3, *p < 0.05, **p < 0.01, ***p < 0.001.).
(3.53 MB TIF)

Video S1 Bright field image of control with no treatment
(1.06 MB AVI)

Video S2 Fluorescence image of control with no treatment
(0.91 MB AVI)

Video S3 Bright field image of embryoid bodies treated with 100 ng/ml of FGF-10
(0.96 MB AVI)

Video S4 Fluorescence image of embryoid bodies treated with 100 ng/ml of FGF-10
(1.39 MB AVI)
