# Supplementary figures and images for: Fibroblast Growth Factor-10 Promotes Cardiomyocyte Differentiation from Embryonic and Induced Pluripotent Stem Cells
Source: PLoS One. 2010 Dec 28;5(12):e14414. doi: 10.1371/journal.pone.0014414 (PMC3011000; doi:10.1371/journal.pone.0014414)

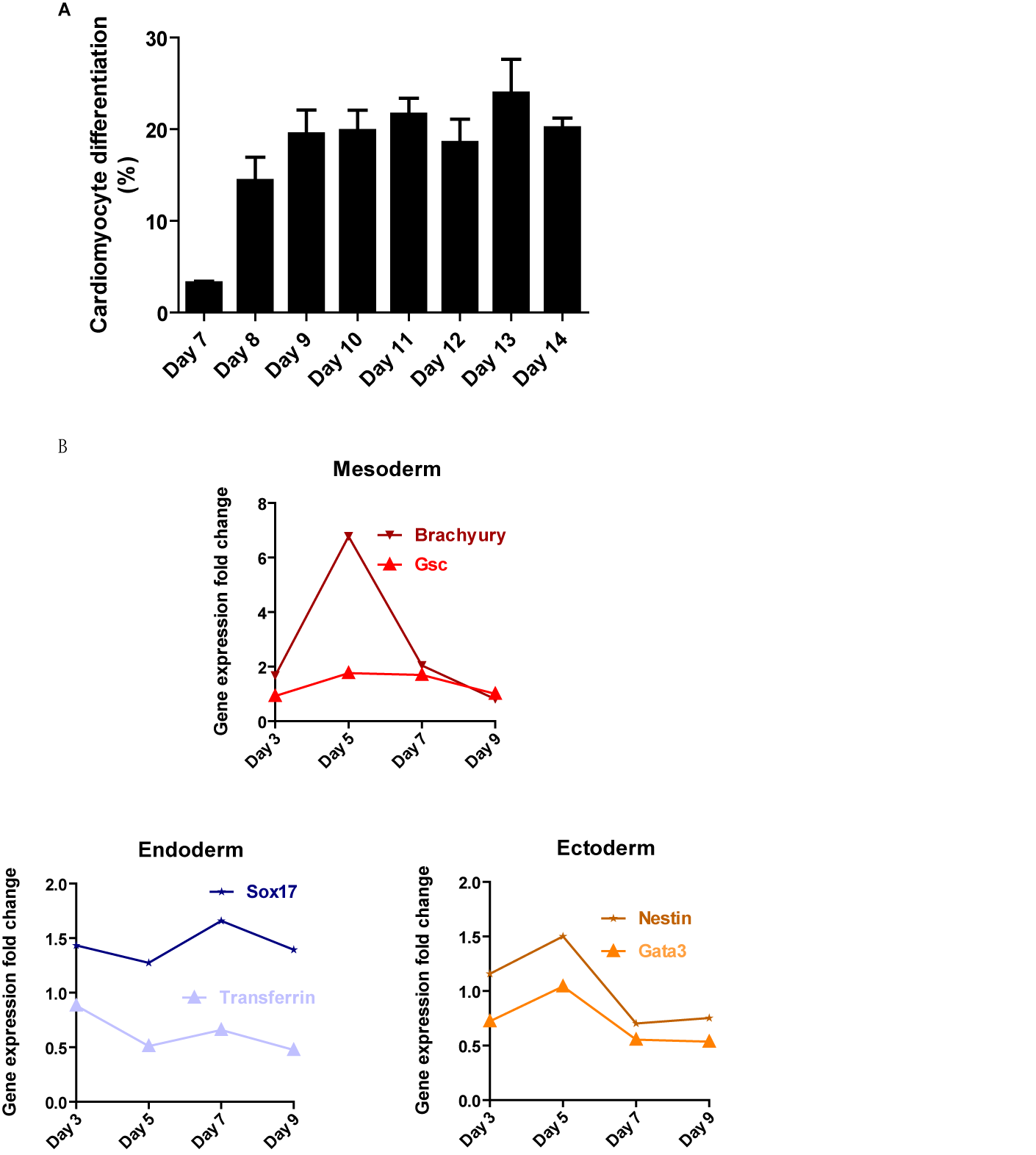

Supplement: Figure S1 — The effect of FGF-10 in controlling ES cell differentiation. A, The percentage of cardiomyocyte differentiation of ES cell treated with FGF-10 over time from day 7 to day 14. B, the time course study of the three germ layer markers upon FGF-10 treatment with quantitative real-time PCR. (6.68 MB TIF) [file pone.0014414.s002.tif]

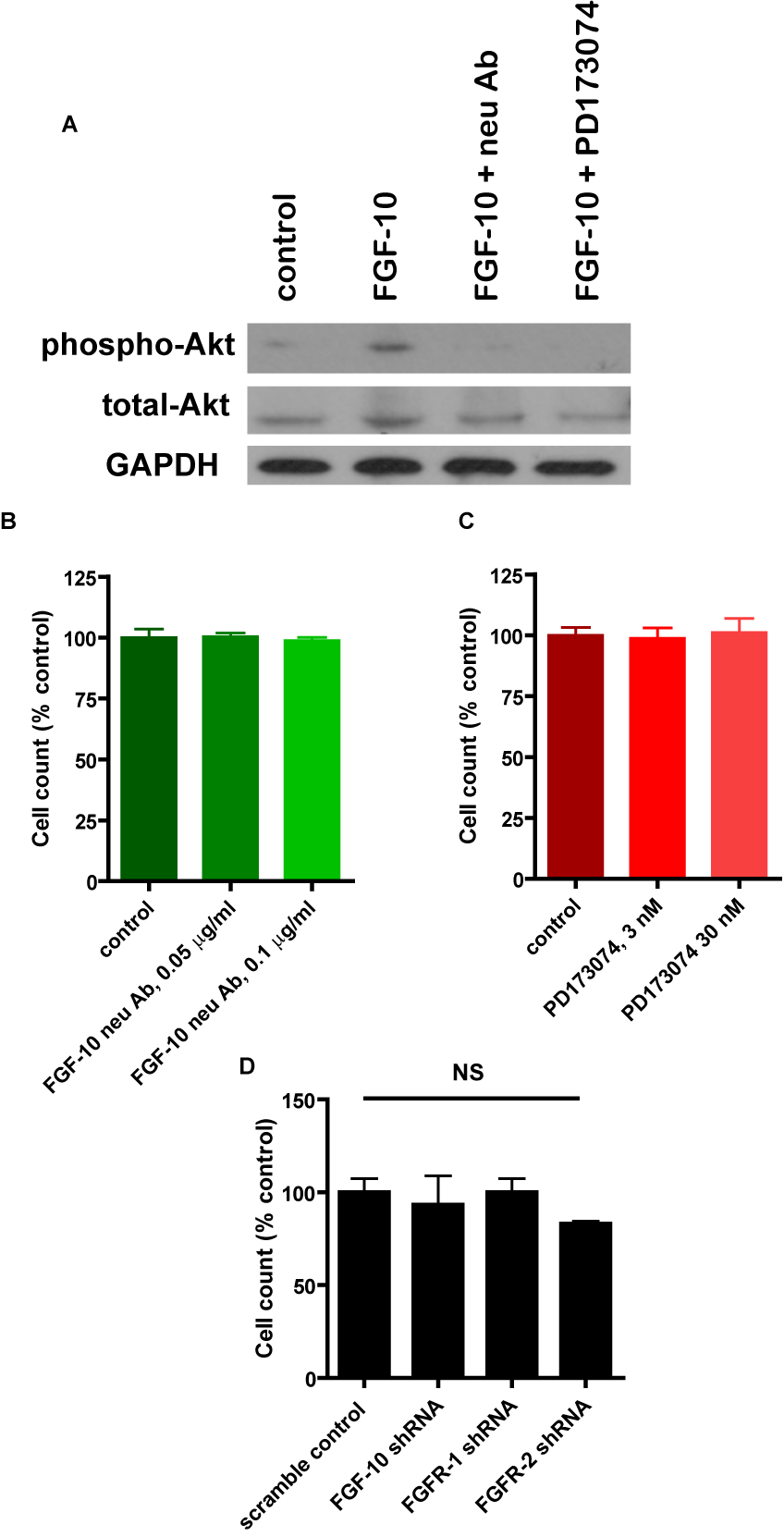

Supplement: Figure S2 — The study of ES cell toxicity under the treatment of FGF receptor inhibitor PD173074. A, Immunoblot analysis of phospho- and total-Akt in embryoid bodies of different treatment. FGF-10, 100 ng/ml. Neutralizing antibody, 0.1μg/ml. PD173074, 30 nM. Control, no treatment. B-D, Total cell count of ES cells treated with an FGF-10 neutralizing antibody, the FGFR inhibitor PD173074, or a lentivirus contained FGF -10, FGFR-1 or FGFR-2 shRNA. Control, no treatment. (4.26 MB TIF) [file pone.0014414.s003.tif]

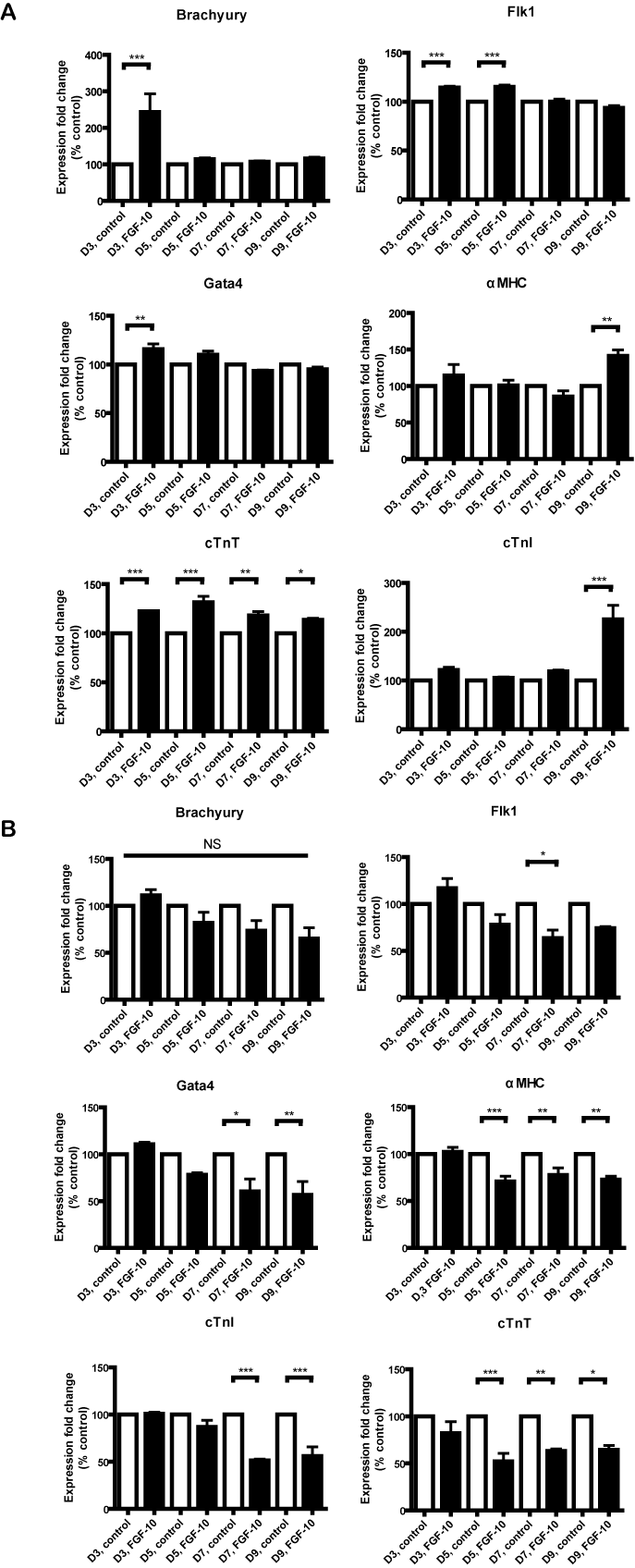

Supplement: Figure S3 — Gene expression profiles of ES cells treated with FGF-10 protein or shRNA. A, RT-PCR results showing the temporal expression pattern of Brachyury, Flk1, Gata4, αMHC, cTnI and cTnT on day 3 (D3), 5 (D5), 7 (D7) and 9 (D9) during hanging drop cultivation of ES cells after the treatment of 100 ng/ml FGF-10. (n≧3, *p < 0.05, **p < 0.01, ***p < 0.001.). B, RT-PCR results showing the temporal expression pattern of Brachyury, Flk1, Gata4, αMHC, cTnI and cTnT on day 3 (D3), 5 (D5), 7 (D7) and 9 (D9) during hanging drop cultivation of ES cells after FGF-10 knockdown by shRNA (n≧3, *p < 0.05, **p < 0.01, ***p < 0.001.). (3.53 MB TIF) [file pone.0014414.s004.tif]
